# Use of private gynaecologist does not relate to better prevention outcomes – An ecological analysis from Finland

**DOI:** 10.1186/1472-6963-6-27

**Published:** 2006-03-06

**Authors:** Elina Hemminki, Tiina Sevon, Kati Tanninen, Eero Pukkala, Ahti Anttila

**Affiliations:** 1 National Research and Development Centre for Welfare and Health (STAKES), P.O. Box 220, 00531 Helsinki, Finland; 2 Finnish Cancer Registry, Institute for Statistical and Epidemiological Cancer Research, Liisankatu 21 B, 00170 Helsinki, Finland

## Abstract

**Background:**

Control of reproduction and prevention of reproductive health problems are important reasons for women to use health services, but the proper organisational level of service provision is not clear. The purpose of this study was to investigate whether visits to private gynaecologists correlate with better health outcomes and worse participation in organised screening for cancer programs.

**Methods:**

This is an ecological analysis using municipalities and groups of women at 5-year age intervals within municipalities as study units. First, the Finnish municipalities (n = 452) were classified into three groups by the age-adjusted level of use of private gynaecologists. Secondly, each age group within municipalities was classified into tertiles by the level of private gynaecologist use. The outcomes were participation in cervical and organised breast cancer screening for cancer programmes, stage of gynaecological and breast cancers at diagnosis, and abortion rates and ratios. All data were obtained from national registers by groups at 5-year age intervals and by municipality. Raw and adjusted (age groups, and in some analyses, municipality social class index) odds ratios, total and by urbanity, were calculated.

**Results:**

The proportions of women participating in cervical cancer and organised breast cancer screening for cancer were somewhat higher in the groups having a low use of private gynaecologists. The proportions of local cancers of all cervical, uterine, ovarian and breast cancers were similar in the three groups, even though the first analysis method suggested somewhat better results for the low-use group in case of cervical cancer and for the high-use group in case of uterine and breast cancer. The rates of induced abortion were higher in municipalities having a high use of private gynaecologists than in those having lower use.

**Conclusion:**

This ecological analysis suggests that frequent use of private gynaecologists relates somewhat to lower organised screening for cancer participation, and is not better in preventing abortions or in detecting cancer earlier. Our results suggest that a planned system relying mainly on general practitioners and public health nurses as the first line care providers is equally good for women's reproductive health as that in which specialists are used.

## Background

Control of reproduction and prevention of reproductive health problems are important reasons for women to use health services. For example, in Finland in the early 1990s, 17% of all outpatient visits of women aged 25–44 years were due to contraception alone [1]. The type of provider consulted is important due to the high volume of visits and the varying orientation and costs of different service providers. In the Finnish context the question is whether these services should be provided by specialists (gynaecologists), general practitioners, public health nurses or midwives. Most women of a reproductive age think that regular check-ups by a gynaecologist are necessary and in 1994 over half (55%) of women had had regular visits in the past five years [1].

In Finland, primary health care is organised by local municipalities in health centres [2,3]. Most health centres are organised by small area responsibility served by general practitioners. Public health nurses, some of whom have a midwifery education, have a large responsibility in preventive health care especially in maternity care and family planning [1,4]. They work relatively independently even though supervised by general practitioners. Finland has had a statutory nationwide organised screening for cancer program by invitation for cervical cancer (PAP-smears) since 1963, and for breast cancer (mammograms) since 1987. The current law (decree) requires the municipalities to invite all women aged 30–60 years to the cervical cancer screening programme, and women aged 50–59 years to the mammography screening programme. Some municipalities have used wider age-limits for breast cancer screening. The interval between invitations is five years for cervical- and two years for breast cancer screening. These organised screening programmes have been demonstrated to be effective [5,6].

In publicly arranged health care, specialists in gynaecology and obstetrics (called gynaecologists subsequently) are available only in hospitals and in their outpatient clinics; gynaecologists can be consulted for problems in an emergency or through referrals. Basic gynaecological problems are treated by general practitioners, and the threshold for specialist consultation vary by practitioner and conditions in the local hospital, especially the length of queues. But many gynaecologists work in private practice, solely or in addition to their hospital work, providing a direct access to specialists.

This privately arranged health care is partly funded by public money through statutory health insurance scheme. All citizens are covered and the money for the insurance is collected by employers and taxes (income based insurance fees). The Insurance covers various sickness related costs, such as sickness day allowances, drug costs, and medical costs in private health care (including physician visits and examinations). Only part of the medical costs is reimbursed. In private health care women can directly seek specialist care. According to reimbursement files, in 1999 23% of women aged 20–69 years visited a private gynaecologist at least once [7]. There is no complete record of the reasons for visiting private gynaecologists, but apparently most of these visits are for preventive purposes, either for contraception or health checks. The need for health check-ups by gynaecologists has been partly created and strengthened by gynaecologists themselves [1].

In the Finnish context, it has been questioned whether such numbers of visits to private gynaecologists are needed, or whether the financial and human resources could be used more efficiently elsewhere in health care. The assumption is that visits provide health benefits, even though at high cost.

A previous study from Finland [7] showed that there was a wide (3.3-fold) variation by region (10 counties) in the use of private gynaecological services. This variation was only partly compensated by use of public services. The purpose of this study is to investigate, whether visits to gynaecologists correlate with better health outcomes and worse participation in organised screening for cancer programs in an ecological analysis using municipalities and groups at 5-year age intervals within municipalities as the study units. The health outcomes available were stage of gynaecological and breast cancers at diagnosis, incidence of cervical cancer, and abortion rates and ratios.

## Methods

Data on gynaecologist visits, screening participation, cancer outcomes and abortions were collected by municipality in 5-year age groups. The number of visits to private gynaecologists, based on reimbursements, was obtained from the Social Insurance Institution of Finland for 1999. There is no formal study on the quality of the register, but it is believed to be relatively complete because it is based on money transfers. The number of visits to public gynaecologists in hospital outpatient clinics by municipality were obtained from a previous study in STAKES (Hospital Benchmarking) [8], based on the routine data collection of hospital districts. Visits due to pregnancy and delivery were excluded. These visits were distinguished by an administrative classification, because the registers do not included the reasons for visits

Data on the numbers of women invited and participating in organised cancer screening in 1998 were obtained from the files of the Mass Screening Registry at the Finnish Cancer Registry [9,10]. Only those age groups in which screening was obligatory for the municipalities were included in this study. Because the data were organised in 5-year-age-groups, 59 years rather than 60 years was used for cervical cancer screening.

The numbers of women diagnosed in 1996–98 to have cervical cancer (C53 in ICD10), uterine cancer (C54), ovarian cancer (C56), and breast cancer (C50) were obtained by stage from the Finnish Cancer Registry. Hospitals, physicians and pathology laboratories are required by law to notify on all cases of cancer. The completeness and accuracy of the register is good [11]. The register classifies cancers into five categories according to stages at diagnosis: localised, regional metastasis, distant metastases, non-localised, NOS (not known whether regional or distant), no information of the stage.

Numbers of induced abortions by municipality, 5-year age groups, and social class in 1998 were obtained from the Finnish Abortion Register [12]. Social class was the one used in the Abortion Register, based on woman's own occupation: upper white collar, lower white collar, workers, enterprisers, students, other. In Finland, STAKES has to be notified of all induced abortions by law. The completeness and data quality in regard to variables used in this study is good [13]. The number of births was obtained from the Finnish birth register [12].

Denominators (the number of women in each age group, by social class in each municipality) were obtained from another project (Jansson, personal communication, STAKES, 2001). Originally the data are from the SOTKA 1999 database, describing the population in December 1998 [14]; SOTKA gets its data from the National Population Register and from Statistics Finland. The social class index was formed by dividing the number of women in the upper and lower white collar classes by the number of women in the class of workers in each age-municipality group; the higher the index, the higher the social class of the population group. The social class in SOTKA is based on various aspects of social class, but women's own occupation is the main determinant. Municipality urbanity (grouped into urban or suburban and rural) was obtained from Statistics Finland.

### Analysis

In the first analysis approach, the municipalities were classified into three groups by the level of use of private gynaecologists: low, middle and high use. The age-adjusted (direct standardisation) rate of private gynaecologist use was calculated separately for each municipality, using the age-distribution of all municipalities as the reference. Municipalities with over 1000 women aged 20–64 years were divided into three groups, each having about same number of women. The limits for age-adjusted rates (visits per woman) turned to be < 0.242, 0.242–0.333 and > 0.333. Municipalities with less than 1000 women aged 20–64 years were then added to the groups having the corresponding rate of visits to the gynaecologist. Similar procedures were used to create three groups by the level of all gynaecologist visits, both private and public. The limits were < 0.579, 0.580–0.687, > 0.687. The women in each group were pooled over the municipalities to avoid the problem of small numbers in small municipalities.

Abortion rates were age- and social class adjusted by the direct standardisation method using the age and social class distributions of all municipalities as the reference. Social class was not available for women with cancer or cancer screening participation, and these outcomes were only age-adjusted.

The correlation between the size of the municipality and the use of private gynaecologist was calculated by Pearson correlation coefficient including all municipalities and by scattergrams between the two, separately for municipalities less then 5000 20–64 women (n = 397 municipalities), 5000–10000 (n = 30), and over 10000 (n = 25). The main results according to municipality size [<5000 (n = 397), 5000–30000 (n = 48) and over 30000 women (n = 7)] were calculated.

In the second analysis approach, each 5-year-interval age-group in each municipality was the unit of analysis. These units were classified into three groups, each having about an equal number of units, by the level of private gynaecologist use. Because different age groups were used for different outcomes, for some municipalities data were lacking and only units having outcome events were included, the number of women as well as the limits of the low, middle and high gynaecologist use vary (see table 3 in Results).

The statistical significance of differences between municipality groups was tested using tests for relative proportions and rate differences by z-test. Odds ratios were calculated by logistic regression and rate ratios by Poisson regression using the lowest private gynaecologist use group as the reference. Furthermore, age-adjusted (5-year interval groups) and age- and social-class-adjusted (index grouped into four classes and as a continuous variable within each 5-year age group) odds ratios were calculated. Similar analyses were made separately for urban/suburban and rural municipalities. SAS GENMOD procedure was used to fit the logistic regression model to allow events per trials responses and to fit Poisson regression model to compare rates.

## Results

Table 1 describes the three groups of municipalities, formed by their use of private gynaecologists. Visits to public gynaecologists were, as a mean, similar in the three groups. Cross-tabulation of the municipality-groups formed by the number of private gynaecologist visits and all gynaecologist visits showed that this was largely also true at municipality level: 72% of municipalities were in the same group according to the two classifications.

### Organised screening for cancer

The proportions of women participating in cervical cancer and organised breast cancer screening were somewhat lower in municipalities having a high use of private gynaecologists, but the differences were small, Table 2. When all gynaecologist visits were inspected, the difference in cervical cancer screening disappeared, but that of breast cancer remained.

When the age groups having similar private gynaecologist use were pooled over the municipalities (the second analysis approach using logistic regression analysis), similar results were obtained for organised cervical cancer screening, showing less participation in the middle and high gynaecology visits groups, Table 3. Adjusting for the municipality social class index diminished the differences between the groups, and in the case of the high-group, it disappeared when the social class index was used as a continuous variable. However, the high social class index and the gynaecologist visit rate strongly correlated with each other.

In the case of organised breast cancer screening, the age-adjusted odds ratios (Table 3) gave similar results in regard to the low and high-use groups as the adjusted percentages in the first analysis approach (Table 2), but the middle-use group had a somewhat higher screening participation than the low-use group. The raw odds ratios were very similar. Adjustment for the municipality social class index increased the difference in the middle-use group (odds ratio 1.13, CI 1.06–1.19) and decreased that of the high-use group (0.84 CI 0.80–0.88).

The analyses were also made separately for urban (including semi-urban) and rural municipalities. The results of urban municipalities were similar to analyses that included all municipalities, but those for rural differed. In the rural areas the participation rate in organised cervical cancer screening was higher in the middle-use group (age-adjusted OR 1.08, CI 1.03–1.14) and the high-use group (OR 1.08; CI 1.02–1.15). In the case of organised breast cancer screening, the differences between the groups were small and statistically non-significant.

### Cancers

The rates of uterine and ovarian cancers among 20–64 years old women were more common, but breast and cervical cancer rates were less common in the low-use group, Table 2. Our assumption was that only the incidence of cervical cancer could be notably influenced by screening or use of gynaecologists.

In the first analysis approach, the proportions of local cancers of all cervical, uterine and ovarian cancers were similar in the three groups. Breast and uterine cancers were found somewhat more often when still localised in the high-use municipalities compared to low or middle use municipalities and cervical cancer was more often localized in the low-use municipalities; but all these differences were small (Table 2). When all visits rather than just private visits were inspected, the cancer outcomes were otherwise similar, but the difference in the proportion of local uterine cancers between the middle- (66%) and high-use (77%) areas were larger (p < 0.05).

In the second analysis approach (when the gynaecologist-visit groups were formed by the 5-year age interval groups rather than using the whole municipality as the unit), all the raw odds ratios suggested that in the high use group cervical cancer was found to be less often localised (OR 0.30, 95% CI 0.17–0.53). When the gynaecologist visits were used as a continuous variable, the result was similar. However, age-adjustment made that difference statistically non-significant (Table 3).

In the case of other cancers, the proportions of localised cancers and raw and age-adjusted odds ratios were similar in the low- and high-use groups. In the middle-use group, ovarian cancers were more often found and breast cancers less often found to be localised, but the findings were not statistically significant. The finding of the first analysis that in the high-use group, breast cancers are found to be more often localised was not confirmed in this second analysis approach. The social class index strongly correlated to the rate of gynaecologist visit, and that analysis was rejected.

### Induced abortions

Abortion rates – even after age and social class standardisation – were higher in municipalities having a high use of private gynaecologists than in those having lower use, Table 4 (the firs analysis approach). When related to the number of births, the difference remained. When all visits were inspected, the differences between the three user-groups were similar.

The second analysis approach (B in Table 4) also showed higher rates and ratios of induced abortions in the group having a high use of private gynaecologists, while those of the middle group were in between. The table also shows the results adjusted for social class, but the high social class index and use of private gynaecologists correlated strongly. The analysis for urban and semi-urban municipalities only showed similar results as those given in Table 4B. In rural municipalities, abortion rates and ratios were similar in the three groups.

### Municipality size

The use of private gynaecologists strongly correlated with the size of the municipality, the high-use municipalities being much larger than the low-use municipalities. Thus, the results by the municipality size were very similar to those by use of private gynaecologists presented in Tables 2, 3 and 4.

## Discussion

Finnish public health care is constructed to serve women's health needs without the necessity for private gynaecologists: organised cancer screening is by invitation in organised programs, primary health care physicians (general practitioners) have catchment areas with referrals to specialist care in outpatient clinics in public hospitals, and preventive care (especially in regard to contraception and pregnancy) was meant to taken care of by the public health nurses and midwives. The public system does not include direct access to a specialist in non-emergency situations. However, this system that was created in 1972 was preceded by a public-insurance-based system, which has coexisted alongside [15]. This has provided women with direct access to directly visit a specialist for even minor issues.

Earlier cancer detection and good contraception have been argued as important reasons for private gynaecologist visits. This ecological analysis suggests that frequent use of private gynaecologists is not important for these aspects in the Finnish context. All the differences found were small and can be due to confounding. There was a weak suggestion that private gynaecologist visits might help in diagnosing breast cancer at an earlier stage. But no such benefit was found for other cancers – for the detection of cervical cancer there was a suggestion of delay – or for participation in organised cancer screening, and the abortion rates were higher in municipalities with many visits. If we forget the potential methodological drawbacks, and assume that the indicators used reflect women's reproductive health more generally, our results suggest that a system relying mainly on general practitioners and public health nurses is at least equally good as that in which specialists are used as the first line care providers.

Certainly there can be benefits to visiting private gynaecologists that were not captured by our indicators. They may include peace of mind (having had a check-up), treatment of infections and advice on healthy habits. These other outcomes, however, do not require the care provider to have medical specialist education. Costs were not studied here, but gynaecologist visits are more expensive due to fees and extra examinations carried out than visits to nurses or general practitioners. We did not have data of reasons for private gynaecologist visits, but earlier surveys as well as everyday observations suggest that most are for preventive purposes.

Our previous analysis showed that visits to public gynaecologists partly compensated, in an ecological analysis, the geographical variation of using private gynaecologists [7]. Studying outcomes by all visits, rather than by private visits alone, gave very similar results. This seems logical because public gynaecologists work mainly by referral. Their major tasks are not preventive, but the treatment of problems, as well as taking care of births.

In Finland two cancers are systematically screened for, cervical and breast cancer. The importance of participation in organised screening for cancer rather than relying on spontaneous examinations is supported by studies showing a smaller risk of cervical cancer after organised screening for cancer [16,17]. It has been suggested that visits to gynaecologists, which often include a PAP-smear and mammogram, might lessen women's interest to participate in organised screening. This study showed that such an impact may exist, but it is not large. Apparently, women using private gynaecologists are health-conscious and participate in screening as well.

There is no organised screening for cancer available for ovarian cancer. The need to regularly visit a gynaecologist has been proposed as necessary for an earlier detection of ovarian cancer. No support for this proposal was found in this study.

The higher abortion rates in municipalities having many visits to private gynaecologists can be due to confounding. The difference was not explained by varying distribution of women's age or social class, but it could be due to other differences between the municipalities, which could not be adjusted for. The municipalities with a high use of gynaecologists were larger than the low-use municipalities, and communal cohesion, for example, might be a confounding factor. Furthermore, the available indicator of social class was crude, possibly resulting in residual confounding by social class.

The ecological approach was chosen purposely. Our data sources, having personal identification codes, could have provided the possibility (at least in theory) of an individual level inspection. However, such an inspection would have been extremely confounded by reasons for visiting a gynaecologist: in addition to the women visiting gynaecologists because of health check-up, women having gynaecological problems would more likely be in the group of private gynaecologist users. In ecological analysis such a bias is lessened. But ecological analysis has its own drawbacks, including the small possibility to control for confounding. The size of municipality and varying social class distributions in the municipalities of different sizes are potential confounders, but on the other hand, the frequency of using private gynaecologists may be a mechanism through which the impact of municipality size or social class has its influence. Thus, we did not adjust for municipality size. Adjustment by the social class index of the municipality usually decreased the difference between the groups, but the social class index and the rate of gynaecologist visits strongly correlated with each other. This supports the hypothesis that they are part of the same chain, and that the use of private gynaecologists is a mechanism through which social class can have an influence.

The health care system – with two-public-channels (municipality-based National Health Service and National Health Insurance) – is unique to Finland. Do our results have any relevance to countries having different health care systems and different cancer screening systems? The question of the most appropriate care provider, both in terms of costs and health outcomes, has been of interest especially in the United States where no uniform health care structure exists, with many different organisations and choice of users [see e.g. [18]]. However, we found no study comparable to our study dealing with reproductive health. Reproductive health – due to both its health and service provision – impacts are important and organising reproductive health services should be based on a well-planned policy. In maternity care, such an organisation exists in most countries, but it should be widened to other areas of reproductive health care as well.

## Conclusion

Our ecological study suggests that a health care system for women's gynaecological health relying mainly on general practitioners and public health nurses is at least equally good than that in which specialists are used as the first line care providers. However, observational studies, including ours are problematic in evaluating health effects of health services. Further research on the usefulness of visits to private gynaecologists is needed, ideally in a trial setting.

## Competing interests

The author(s) declare that they have no competing interests.

## Authors' contributions

EH, EP and AA designed the study; TS and KT collected the data; TS, KT and EP conducted the data analysis; EH drafted the paper; TS, EP and AA critically revised the paper.

## Pre-publication history

The pre-publication history for this paper can be accessed here:



## Figures and Tables

**Table 1:** Description of municipalities having low, middle and high rates of private gynaecologist visits, Finland 1999.

	Low <0.242	Middle 0.242–0.333	High >0.333	Total
Number of municipalities	285	109	58	452
Number of women 20–64 years	558058	514861	472230	1545149
Mean number of women 20–64 years	1958	4724	8142	3418
Median number of women 20–64 years	1186	1639	1617	1289
Age-adjusted rate of private gynaecologist visits per 100 women^1)^	16.4	29.1	39.3	27.6
Age-adjusted rate of public gynaecologist visits per 100 women^1)^	33.1	32.9	34.1	33.3
Age-adjusted rate of total gynaecologist visits per 100 women^1)^	49.4	62.0	73.3	60.8

^1) ^The number of visits and women aged 20–64 years were pooled in each category (low, middle, high).

**Table 2:** Proportions (%) and age adjusted odds ratios of women participating in cervical cancer screening (30–59-years-old) and breast cancer screening (50–59-years-old) in 1998 and having cancer (per 10 000), proportions and age-adjusted odds ratios for having localized cancers among 20–64-year- old in 1996–1998 in the three groups of municipalities, formed by the rate of private gynaecologist visits per woman in 1999.

	Low < 0.24	Middle 0.24–0.35	High > 0.35	Total
Cervical cancer screening (invited, n)	(79071)	(71598)	(66382)	(217051)
participating, raw %	75.4	74.7**	71.6***	74.0
participating, age-adjusted % ^1)^	74.7	74.6	72.0***	74.0
Breast cancer screening (invited, n)	(58857)	(55041)	(47718)	(161616)
participating, raw %	90.3	88.0***	84.5***	87.8
participating, age-adjusted % ^1)^	90.3	88.1***	84.5***	87.8
Cervical cancer (number of cancers)	(692)	(833)	(664)	(2189)
(having cancer per 10 000)	(12.5)	(16.2***)	(14.1**)	(14.2)
local cancer, age-adjusted % ^1)^	94.8	94.3	93.2*	94.2
Uterine cancer (number of cancers)	(364)	(295)	(265)	(924)
(having cancer per 10 000)	(6.6)	(5.7)	(5.6*)	(6.0)
local cancer, age-adjusted % ^1)^	70.9	69.8	75.5*	71.9
Ovarian cancer (number of cancers)	(374)	(312)	(291)	(977)
(having cancer per 10 000)	(6.8)	(6.1)	(6.2)	(6.3)
local cancer, age-adjusted % ^1)^	48.2	45.7	48.5	47.3
Breast cancer (number of cancers)	(2047)	(2085)	(2013)	(6145)
(having cancer per 10 000)	(37.0)	(40.5**)	(42.6***)	(39.9)
local cancer, age-adjusted % ^1)^	55.8	55.5	61.2***	57.5

^1) ^Calculated by using the age-distribution of all municipalities as the reference.

The statistical significance in the proportions and rate differences was calculated by separately comparing the middle and high groups to the low group. * = p < 0.05, ** = <0.01, *** = p < 0.001.

**Table 3:** Age-adjusted odds ratios (95% confidence intervals, CI) of participation in cervical cancer (30–59-year-olds) and breast cancer screening (50–59-year-olds) in 1998 and proportions (%) of localised cancers among 20–64-year olds (1996–1998) in three groups, formed by the rate of private gynaecologist visits in 5-year-age groups in 1999 ^1)^.

	Low ^3)^	Middle	High
Cervical cancer screening (n = 2543) ^2)^			
(Gynaecologist visit rate, per 100)	(< 0.17)	(0.17–0.28)	(< 0.28)
(Number of women)	(31138)	(52481)	(122585)
% participating	74.2	71.7	71.7
Age-adjusted OR (95% CI)	1.00	0.85 (0.82–0.88)	0.79 (0.77–0.81)
Breast cancer screening (n = 833)			
(Gynaecologist visit rate per 100)	(<20)	(20–29)	(>29)
(Number of women)	(24163)	(30960)	(106473)
% participating	90.0	90.5	86.6
Age-adjusted OR (95% CI)	1.00	1.06 (1.00–1.12)	0.73 (0.70–0.76)
Cervical cancer (n = 852)			
(Gynaecologist visit rate per 100)	(<16)	(16–27)	(>27)
(Number of cancers)	533	723	930
Local, raw %	97.2	96.0	91.2
Age-adjusted OR (95% CI)	1.00	0.87 (0.44–1.72)	0.61 (0.33–1.15)
Uterine cancer (n = 486)			
(Gynaecologist visit rate per 100)	(<18)	(18–30)	(>30)
(Number of cancers)	192	272	459
Local, raw %	71.4	70.6	73.0
Age-adjusted OR (95% CI)	1.00	0.97 (0.65–1.46)	1.10 (0.75–1.62)
Ovarian cancer (n = 575)			
(Gynaecologist visit rate per 100)	(<19)	(19–30)	(>30)
(Number of cancers)	213	253	511
Local, raw %	44.6	51.4	46.4
Age-adjusted OR (95% CI)	1.00	1.44 (0.98–2.12)	1.19 (0.84–1.68)
Breast cancer (n = 1519)			
(Gynaecologist visit rate per 100)	(<18)	(18–29)	(>29)
(Number of cancers)	809	1551	3781
Local, raw %	57.6	53.3	59.2
Age-adjusted OR (95% CI)	1.00	0.85 (0.72–1.01)	1.07 (0.92–1.26)

^1) ^The age groups with similar visit rates were pooled over the municipalities into three groups, see Methods, the second analysis approach. The lowest use group is the reference group. The limits of groups (gynaecologist visits per woman) vary by outcome due to different age-groups and varying lacking data in different analyses.

^2) ^Each unit = one 5-year-interval age-group in each municipality

^3) ^The reference group for calculating age-adjusted odds ratios by logistic regression.

**Table 4:** Abortion rates (per 1000) and mean abortion/birth ratios (per 100) among 20–44-year-old women in 1998 in the three groups of municipalities, formed by the rate of private gynaecologist visits in 1999 (A), and in the three groups formed by the rate of private gynaecologist visits in groups at 5-year age intervals in 1999 (B) ^1)^.

A	Low	Middle	High
	< 24.2%	24.2–33.3%	> 33.3%
(Number of women)	(300278)	(291580)	(278044)
Raw abortion rate	8.1	10.2***	11.4***
Rate difference (95% CI)		2.14 (1.65–2.63)	3.32 (2.81–3.83)
Age-adjusted rate^2)^	8.4	10.2	11.1
Rate difference (95% CI)		1.80 (1.51–2.09)	2.72 (2.43–3.01)
Age and social class adjusted rate ^2)^	8.2	10.9	11.0
Rate difference (95% CI)		2.66 (2.37–2.95)	2.76 (2.47–3.05)
(Number of births)	(19710)	(18623)	(16496)
Age-adjusted abortion-birth ratio	12.1	16.0	19.4
Ratio difference (95% CI)		3.85 (3.45–4.26)	7.30 (6.88–7.72)
B			
	< 11%	11–22%	> 22%
(Number of women)	(153064)	(265114)	(450412)
Raw abortion rate	10.6	10.8	9.0
Age-adjusted rate ratios (95% CI) ^3)^	1.00	1.16 (1.09–1.23)	1.34 (1.25–1.43)
Age- and social-class-adjusted rate ratios (95% CI) ^3)^	1.00	1.11 (1.04–1.19)	1.23 (1.14–1.33)
(Number of births)	(11715)	(16989)	(26035)
Abortion-birth ratio	13.8	16.8	15.6
Age-adjusted rate ratios (95% CI) ^3)^	1.00	1.35 (1.25–1.46)	1.59 (1.47–1.73)
Age- and social-class-adjusted rate ratios (95% CI) ^3)^	1.00	1.25 (1.16–1.35)	1.40 (1.28–1.53)

^1)^The age groups with similar visit rates were pooled over the municipalities into three groups, see Methods. The lowest use group is the reference group.

^2) ^Calculated by using the age-distribution and social class of all municipalities as the reference.

^3) ^Calculated by Poisson regression, the reference group is "Low".
